# Climate change impacts on human health over Europe through its effect on air quality

**DOI:** 10.1186/s12940-017-0325-2

**Published:** 2017-12-05

**Authors:** Ruth M. Doherty, Mathew R. Heal, Fiona M. O’Connor

**Affiliations:** 1 School of GeoSciences, University of Edinburgh, Alexander Crum Brown Road, Edinburgh, EH9 3FF UK; 2 School of Chemistry, University of Edinburgh, David Brewster Road, Edinburgh, Scotland EH9 3FJ UK; 3 Met Office Hadley Centre, FitzRoy Road, Exeter, EX1 3PB UK

**Keywords:** Climate change and air pollution, Ozone, Particulate matter, Human health

## Abstract

This review examines the current literature on the effects of future emissions and climate change on particulate matter (PM) and O_3_ air quality and on the consequent health impacts, with a focus on Europe. There is considerable literature on the effects of climate change on O_3_ but fewer studies on the effects of climate change on PM concentrations. Under the latest Intergovernmental Panel on Climate Change (IPCC) 5th assessment report (AR5) Representative Concentration Pathways (RCPs), background O_3_ entering Europe is expected to decrease under most scenarios due to higher water vapour concentrations in a warmer climate. However, under the extreme pathway RCP8.5 higher (more than double) methane (CH_4_) abundances lead to increases in background O_3_ that offset the O_3_ decrease due to climate change especially for the 2100 period. Regionally, in polluted areas with high levels of nitrogen oxides (NO_x_), elevated surface temperatures and humidities yield increases in surface O_3_ – termed the O_3_ climate penalty – especially in southern Europe. The O_3_ response is larger for metrics that represent the higher end of the O_3_ distribution, such as daily maximum O_3_. Future changes in PM concentrations due to climate change are much less certain, although several recent studies also suggest a PM climate penalty due to high temperatures and humidity and reduced precipitation in northern mid-latitude land regions in 2100.

A larger number of studies have examined both future climate and emissions changes under the RCP scenarios. Under these pathways the impact of emission changes on air quality out to the 2050s will be larger than that due to climate change, because of large reductions in emissions of O_3_ and PM pollutant precursor emissions and the more limited climate change response itself. Climate change will also affect climate extreme events such as heatwaves. Air pollution episodes are associated with stagnation events and sometimes heat waves. Air quality during the 2003 heatwave over Europe has been examined in numerous studies and mechanisms for enhancing O_3_ have been identified.

There are few studies on health effects associated with climate change impacts alone on air quality, but these report higher O_3_-related health burdens in polluted populated regions and greater PM_2.5_ health burdens in these emission regions. Studies that examine the combined impacts of climate change and anthropogenic emissions change under the RCP scenarios report reductions in global and European premature O_3_-respiratory related and PM mortalities arising from the large decreases in precursor emissions. Under RCP 8.5 the large increase in CH_4_ leads to global and European excess O_3_-respiratory related mortalities in 2100. For future health effects, besides uncertainty in future O_3_ and particularly PM concentrations, there is also uncertainty in risk estimates such as effect modification by temperature on pollutant-response relationships and potential future adaptation that would alter exposure risk.

**Electronic supplementary material:**

The online version of this article (10.1186/s12940-017-0325-2) contains supplementary material, which is available to authorized users.

## Background

The World Health Organization (WHO) has reported strengthened evidence for adverse health effects related to exposure to particulate matter (PM), ozone (O_3_) and nitrogen dioxide (NO_2_) [1]. Their evidence comes from various types of epidemiological studies [2]. For PM, both short-term and long-term exposure to PM_2.5_ (particle diameter < 2.5 μm) is associated with all-cause and cardiovascular mortality and morbidity. Evidence now also links long-term exposure with other health outcomes including adverse birth outcomes and childhood respiratory disease [1]. There is also strengthening evidence for short-term effects on mortality and morbidity from the larger particle size fractions PM_10_ (diameter < 10 μm).

For O_3_, short-term exposure is associated with all-cause, cardiovascular and respiratory mortality, and respiratory and cardiovascular hospital admissions. There is now strengthened evidence for effects of long-term exposure to O_3_ on respiratory and cardiorespiratory mortality in warm season months [1], although taken as a whole this evidence is limited at present, as it is primarily derived from studies in North America [3]. For both PM and O_3_ there are indications that adverse effects exist down to low concentrations, such that it is hard to discern any threshold concentration. Whilst evidence has also strengthened for independent impacts of both short-term and long-term exposure to NO_2_ on mortality, hospital admissions and respiratory symptoms, because of strong correlations between NO_2_ and other air pollutants it remains difficult to discern a direct effect [1]. This, coupled with the current evidence that for both PM and O_3_ adverse effects exist down to low concentrations, means that this review focuses predominantly on these two pollutants: O_3_ and PM.

Emissions of the main air pollutant precursors: primary PM, black carbon (BC); nitrogen oxides (NO_x_); sulphur dioxide (SO_2_), carbon monoxide (CO), methane (CH_4_) and non-methane volatile organic compounds (NMVOCs) in Europe have declined in the last decade or so, resulting in improvements in air quality across the region [4]. However, due to the complexities of the processes linking emissions and air quality, notably interactions with meteorology, reductions in local or regional emissions do not always produce a reduction in atmospheric concentrations. Hence air pollution remains a high profile issue, now and for the future.

This review examines the current evidence for the effects of climate change on ambient air quality and on the consequent health impacts over Europe. The topics covered in this review are as follows. First, current health burdens and risk estimates are outlined as well as health impacts from the well-documented 2003 heatwave over Europe. The sensitivity of O_3_ and PM to changes in climate and in climate extremes and the key driving processes are then reviewed. Air quality and health impacts associated with recent IPCC scenarios for a) future climate and with b) combined future climate and emissions are then described and key non-climate/emissions determinants of future air quality highlighted. Finally, this review presents conclusions and knowledge gaps.

## Review

### Current health burdens and risk estimates

The estimated health burdens attributable to ambient air pollution are substantial. The Global Burden of Disease project estimated that exposure to PM_2.5_ in 2015 contributed 4.2 million deaths globally, whilst exposure to O_3_ contributed 0.25 million deaths globally [5]. Across the European Union (EU), the European Environment Agency (EEA) estimated that about 467,000 people died prematurely in 2013 due to long-term exposure to ambient PM_2.5_ [4]. For O_3_, the EEA estimated that short-term exposures in 2013 contributed to more than 17,000 premature mortalities [4]. This assumes a threshold concentration for effect of 35 ppbv (70 μg m^−3^). Whilst the proportions of the EU urban population exposed to ambient PM concentrations exceeding EU limit or target values have declined in the last decade, they have varied for exposure to O_3_ partly due to year-to-year variability in meteorology [4, 6].

Dose-response coefficients used to quantify the risk of mortality related to short and long-term exposure to PM_2.5_ and O_3_ are given in Table 1. For short-term exposure, these coefficients are mainly based on the Health Risks of Air Pollution In Europe (HRAPIE) WHO project [7]; for O_3_ results from the Air Pollution and Health: A European and North American Approach APHENA study [8] and the UK Committee on the Medical Effects of Air Pollution (COMEAP) meta-analysis [3] are also provided. For long-term exposure, findings from long-term American Cancer Society (ACS) cohort studies are given [9, 10]. For long-term exposure to PM_2.5_, the WHO [7] suggest an increased premature mortality risk of 6.2% per 10 μg m^−3^ exposure measured using annual-mean PM_2.5_ concentrations (Table 1). For short-term exposure to O_3_, an increased premature mortality risk of 0.29% per 10 μg m^−3^ exposure measured using daily maximum 8-h running mean O_3_ is suggested by the WHO [7] (Table 1); they also recommend a threshold of 70 μg m^−3^ (35 ppbv) due to greater data availability for the warm season.

**Table 1:** Risk estimates or dose-response coefficients for short and long-term exposure to ambient PM_2.5_ and O_3_

Exposure and Reference	Outcome	Risk estimate	95% Confidence interval	Metric
Short term PM_2.5_ [7]	All cause	1.2% per 10 μg m^−3^	0.45, 2.0%	Daily mean
Long term PM_2.5_ [9]	All cause	0.3% per 1 μg m^−3^	0.1, 0.5%	Annual mean
Long term PM_2.5_ [7]	All cause	6.2% per 10 μg m^−3^	4.0, 8.3%	Annual mean
Short term O_3_ [8]	All cause	0.18% (EU) per 10 μg m^−3^	0.07, 0.30% (EU)	Daily mean O_3_
		0.31% (USA) per 10 μg m^−3^	0.09, 0.52% (USA)	
Short term O_3_ [7, *3*]	All cause	0.29% per 10 μg m^−3^	0.14, 0.43%	Daily maximum 8-h O_3_
		*0.34% per 10 μg m* ^*−3*^	*0.12, 0.56%*	
Long-term O_3_ [7] –based on [10]	Respiratory mortality	1.4% per 10 μg m^−3^	0.5, 2.4%	Daily maximum 8-h O_3_ [April-Sept]

## Current air pollution and health impacts - the 2003 heatwave analogue

Many interacting processes control the concentrations of O_3_ and PM at a given location: emissions, transport, transformation and deposition, all of which can be affected directly or indirectly by meteorology and climate. O_3_ has an average tropospheric lifetime of about a month [11]. The different components of PM have varying lifetimes but typical values are around ~1–2 weeks [12]. Horizontal transport times across the mid-latitudes are ~2 weeks [13], hence both O_3_ and PM can be transported across continents.

Elevated air pollution associated with extreme events such as summer heat waves has received much attention in the literature. Patz et al. [14] found heatwaves can often be associated with O_3_ exceedance days. In particular, the European heat wave of 2003 and the accompanying high O_3_ levels have been studied in detail [15–18]. Relevant processes identified include: persistent high-pressure systems and extended residence time in the atmospheric boundary layer, extensive forest fires, enhanced levels of natural biogenic isoprene (an O_3_ and PM precursor) and suppressed dry deposition of O_3_. Wildfires have also been associated with high PM_2.5_ levels [19]. High levels of O_3_ and PM_10_ are estimated to have contributed to one-third of the excess deaths occurring during the 2003 heatwave in the UK [20, 21]. In the Netherlands, Fischer et al. [22] estimated an excess of 1000–1400 deaths, with 400–600 of these deaths related to O_3_ and PM_10_. For nine cities in France (where the impacts of the 2003 heatwave were largest), Filleul et al. [23] estimated that the excess risk of death was significant (1.01%; 95% CI, 0.58–1.44) for an O_3_ increase of 10 μg m^−3^, and highlighted the joint risk due to temperature and O_3_. Studies suggest that summer 2003 could be a normal summer in the coming decades under climate change [24, 25]. Indeed, Stott et al. [26] estimated with >90% confidence that human influence has at least doubled the risk of a heatwave equivalent to that experienced across Europe in 2003. A detailed description of the influences of meteorological and climate variables in relation to climate change effects on O_3_ and PM concentrations is given below.

## Future air quality and health impacts

Future air quality depends on several factors: anthropogenic emissions, natural emissions (that are climate sensitive), as well as climate change that results from changes in emissions of long-lived greenhouse gases. Future emission scenarios generally provide emission trajectories for both greenhouse gases used for climate projections as well as emissions for primary PM and precursor emissions of surface O_3_ and secondary PM. Hence, emissions and climate change are linked in this context of future air quality. In the following sections, first the effect of climate change including climate extremes on air quality is reviewed, and recent estimates of changes in air quality using the latest IPCC scenarios presented. Subsequently, studies that examine the impacts of the combination of climate and emissions changes on air quality and on human health burdens are featured. Absolute changes in future O_3_ or PM levels are discussed from multi-model estimates or from the most recent literature.

## The impacts of climate change and change in climate extremes on O_3_ and PM air pollution

**Fig. 1:**
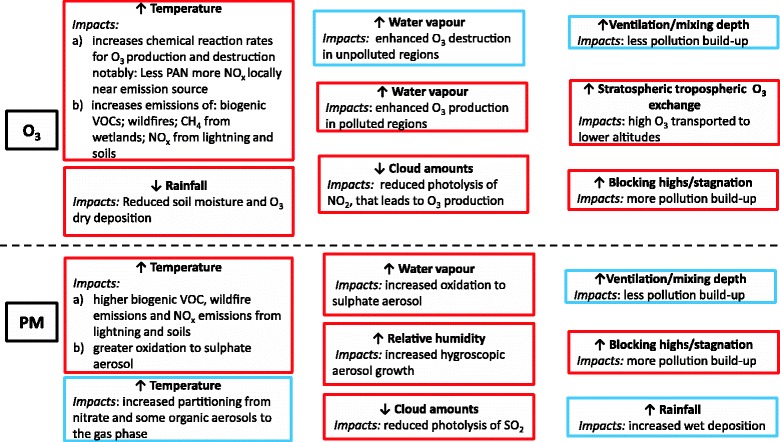
The impacts of meteorological and climate variables on O_3_ and PM concentrations as determined from studies in the literature. ↑ represents an increase in the variable and↓ a decrease. A Red (blue) box outlines depict where changes in a given variable predict an increase (decrease) in O_3_ or PM

The impacts of important meteorological and climate variables on surface O_3_ and PM, are summarised in Fig. 1, which is based on comprehensive reviews [27–29] as well as studies outlined below. The key processes that influence air quality occur through changes in temperature, water vapour, precipitation and clouds, and meteorological transport and mixing. Climate variables not only influence air pollutant concentrations but they can also act as confounding and modifying factors of air pollution concentration-health response relationships. For example, Pattenden et al. [30] showed that adverse health effects of exposure to O_3_ were greater on the hottest days.

O_3_ is strongly correlated with temperature [31] due to associations of increased temperature with enhanced O_3_ photochemical reaction rates, as well as with stagnation events (discussed below), and with elevated natural emissions from biogenic and wild fire sources. In particular, several studies highlight that higher temperatures enhance rates of peroxyacetylnitrate (PAN) decomposition [32–34] leading to local NO_2_ and O_3_ increases in polluted emission regions. A number of these factors were also established as the cause of high levels of O_3_ during the 2003 heatwave (see above). Natural emissions of isoprene strongly increase with increasing temperature (but also decrease with increasing atmospheric carbon dioxide CO_2_). Several authors have highlighted the crucial role of the temperature sensitivity of isoprene emission, finding this to be the dominant mechanism for increasing O_3_ levels in polluted regions [33, 34]. However, CO_2_ inhibition of isoprene emissions in a future higher CO_2_ climate may offset temperature-driven emission increases [35–40]. In addition, Ito et al. [41] and Fiore et al. [29] suggest the O_3_ response to temperature depends on the amount of recycling of NO_x_ from isoprene nitrates. Sustained elevated temperatures can lead to reduced soil moisture, which decreases dry deposition of O_3_ through plant stomata, and to increased wildfires which contribute to O_3_ precursor emissions. Another important global O_3_ precursor, methane (CH_4_), has a large natural emissions source from wetlands; these emissions are likely to increase under climate change along with CH_4_ emissions from other sources as a result of climate feedbacks (e.g. permafrost thaw) [42]. Forkel and Knoche [43] also suggest that elevated temperatures increase soil NO_x_ emissions, which they found increased summer daily maximum surface O_3_ concentrations slightly in agricultural areas in Europe.

The overall effect of temperature-driven processes on PM is even more complex to disentangle than for O_3_ due to opposing influences on various PM components [29]. Elevated temperatures enhance sulphur dioxide (SO_2_) oxidation to sulphate aerosol [44, 45], and increase partitioning to the gas phase which reduces nitrate and some organic aerosol species [28, 29, 46, 47]. In particular, several studies over the USA suggest large decreases of nitrate PM with increasing temperature, and this is the dominant effect on PM concentrations in regions where nitrate is a relatively large component [28, 48], as is often the case in urban European regions. Large changes in PM are possible due to elevated emissions from biogenic and wild fire sources, yielding carbonaceous particles, mineral dust and secondary organic aerosol [29].

Specific humidity also plays an important role in chemistry as higher atmospheric water vapour (associated with elevated temperature) will increase O_3_ destruction in low-NO_x_ environments e.g. [49], causing a reduction in ‘background’ concentrations of surface O_3._ However, higher water vapour also leads to higher HO_x_ (OH + HO_2_) that enhances O_3_ in high NO_x_ environments [28, 50]. For PM, increased water vapour alters aerosol properties and hygroscopic growth and through higher OH levels enhances SO_2_ oxidation leading to higher particle sulphate concentrations e.g. [45, 50]. Particle nitrate levels also increase with higher humidities [48]. Rainfall frequency and amount affects wet deposition processes that remove pollutants from the atmosphere. PM concentrations decrease in areas simulated to have increased precipitation frequency and vice versa [51–53]. Under climate change it is generally thought that wetter regions of the world will get wetter and drier regions drier [54, 55]. Cloud amount also affects the amount of incoming solar radiation and hence photolysis rates that influence surface O_3._ Several authors report increased summer surface O_3_ concentrations over Europe and the NE USA due to reduced cloud amounts that lead to enhanced photolysis rates [56–58], particularly that of NO_2_ which favours O_3_ formation [43].

Meteorological transport and mixing, in particular wind speed and direction and boundary-layer height, determines the dispersion, deposition or stagnation of pollutants and their precursors. However, change in many of these variables e.g. wind speed cannot be reliably predicted by global or regional climate models due to their dependence on small-scale features such as topography. Under climate change, convection is expected to be deeper, although less frequent [59, 60]. Lightning NO_x_ emissions typically increase e.g. [11] although this finding may be sensitive to the model lighting scheme used [61]. Likewise, climate change is predicted to increase stratosphere-troposphere exchange as a result of an enhanced Brewer Dobson circulation [62] which increases the stratospheric contribution to surface O_3_ [63, 64]. Studies provide inconsistent results on the impact of climate change on mixing depth, with increases and decreases in different regions [52, 65–67].

Changes in climate and in climate extremes also have the potential to alter transport pathways e.g., [65, 68], which are influenced by the dominance and passage of both low and high pressure systems. For example, Glotfelty et al. [69] suggest that climate change, through an enhanced low pressure centre over eastern Russia increases intercontinental transport of air pollution from the region increasing global average O_3_ and PM_2.5_. However, Doherty et al. [34], using a passive tracer approach, found that shifts in transport patterns due to climate change are unlikely to have a major role in influencing the annual-mean O_3_ response, but may be important when considering changes in O_3_ metrics influenced by extreme values such as daily 8-h maximum surface O_3_. In particular, the frequency and magnitude of O_3_ and PM pollution episodes will likely be affected by climate change through changes in the frequency of low pressure system passage [68, 70] or the frequency of blocking episodes. Multi-model studies show a consistent decrease in winter and summer blocking over Europe in the twenty-first century [71]. However, in summer the accompanying poleward shift of the storm track into the region of frequent high-latitude blocking, may lead to a greater number of storms obstructed by blocking high pressure systems in the future [71]. Changes in large-scale blocking may affect local stagnation and heatwave episodes. However the relationship between blocking and stagnation remain unclear [72]. Indeed, Horton et al. [73] report an increase in annual-mean and springtime stagnation occurrences over Mediterranean Europe by the late twenty-first century. The authors noted that biases in modelled surface wind speeds used to create their air stagnation index were large. Overall, Kirtman et al. [74] concluded that evidence and agreement on the impact of climate change on transport pathways are still lacking.

## Future climate scenarios and air quality impacts

**Fig. 2:**
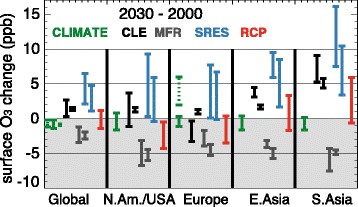
Reproduction (with permission) of Figure 11.22 from chapter 11, IPCC WG1 Fifth Assessment Report (Kirtman et al. [74]) showing changes in surface O_3_ (ppb) between year 2000 and 2030 driven by climate alone (CLIMATE; green) or emissions alone following CLE (black), MRF (gray), SRES (blue) and RCP (red) emission scenarios. Bars represents multi-model standard deviation with the exception of the green dotted bar over Europe, which represents the range of climate-only changes in summer daily maximum O_3_ from a single-model study [43]. For further details, see Kirtman et al. [74]

Estimates of the global and regional average surface O_3_ response to climate change alone between 2000 and 2030 based on the review findings of Fiore et al. [29] for several greenhouse gas emission scenarios including the Special Report on Emission Scenarios (SRES) [75] and the latest IPCC Representative Concentration pathways (RCP) scenarios are depicted in Fig. 2 [74]. The ranges reflect multi-model differences in spatial averages (solid green lines) and spatial variability within a single model (dashed green lines). The global–mean temperature change projected with Global Climate Models (GCMs) driven by the SRES climate scenarios is 1.4–6.3 °C in the 2090s. GCM projections of global average warming using the RCP climate scenarios is between 0.3–1.7 °C for RCP 2.6 (the least extreme pathway representing 2.6 W m^−2^ net radiative forcing at 2100) and 2.6–4.8 °C for RCP 8.5 (the most extreme scenario) in 2100 compared to 1986–2005 [76].

Typically, larger O_3_ changes occur due to changes in emissions of short-lived O_3_ precursors alone as compared to climate change alone in the 2030s (Fig. 2). The decrease in global mean O_3_ is driven by higher water vapour and temperatures. The higher temperatures can lead to local O_3_ increases during the peak pollution season (2–6 ppbv) for Central Europe (green dashed line based on [43]). Overall, a warmer, moister climate increases O_3_ in polluted regions [74], the so-called “climate penalty effect” [68, 77–79].

Over the longer time frame out to 2100, studies of climate change impacts on air quality suggest the same effects on background O_3_ and on O_3_ in polluted regions as described above, but the impact is typically larger with the larger climate signal. In a multi-model study, Doherty et al. [34] suggest annual-mean O_3_ increases of up to 6 ppbv in polluted regions reaching 14 ppbv in the season of maximum O_3_ under the SRES A2 greenhouse gas or climate scenario. In a regional European multi-modelling study to 2050 using the SRES A1B climate scenario, Langner et al. [80] report that, in southern Europe, climate change leads to increased summer mean O_3_ (0–3 ppbv) and increased summer daily maximum O_3_ (3–6 ppb). In northern Europe, they found reductions (0–3 ppbv) for both mean and daily maximum O_3_ in summer. Collette et al. [79] also found similar geographical patterns of projected changes in O_3_ with an increase over southern continental Europe and a decrease over northern Europe and the British Isles. Several studies report climate change to have a greater impact on episodic O_3_ (e.g., the 95th percentile of hourly O_3_) than on longer-term (e.g., summer-mean) averages [50, 81]).

Most recently, several studies have suggested a PM climate penalty in the future [45, 53]. A PM climate penalty simulated in 2050 and 2100 in the eastern USA was attributed to enhanced sulphate concentrations associated with higher temperatures and humidities [45]. A recent multi-model study suggested that climate change, simulated under the RCP 8.5 climate scenario, increases the aerosol burden and surface PM concentrations, through a reduction in large-scale precipitation over northern mid-latitude land regions [53]. Over Europe, one regional modelling study reported the geographical patterns of the impact of climate on surface summer PM levels to be less robust than for O_3_ [82].

There is high agreement from modelling studies projecting increases in the frequency and duration of extreme O_3_ pollution events but there is large variability in predicted spatial pattern and incidence of these events [28, 29]. However, the collective evidence suggests that increasing temperatures during air stagnation episodes in polluted environments will increase peak pollution [28, 29].

There have been only a few studies to date examining the effect of climate change alone on human health burdens from air quality; for PM_2.5_ studies have focussed on long-term exposure, whilst for O_3_ studies have considered either short-term or long-term exposure. Fang et al. [50] quantified the effect of climate change induced changes in PM_2.5_ and O_3_ air quality over the twenty-first century (2090s–1990s) under the moderate SRES A1B scenario on global premature mortalities due to long-term exposure. PM_2.5_ concentrations increases due to elevated sulphate concentrations and reduced precipitation over the major emission source regions, led to an increase in global annual premature mortality associated with chronic exposure to PM_2.5_ of approximately 100,000 deaths (95% confidence interval, CI, of 66–130,000) with corresponding years of life (YLL) lost increasing by nearly 900,000 (95% CI, 576,000–1,128,000) years [50]. Higher O_3_ in polluted regions also increased annual premature mortality due to respiratory disease from chronic O_3_ exposure by 6,300 deaths (95% CI, 1600–10,400) [50]. On average, across 50 U.S. cities, higher O_3_ under the SRES A2 high climate scenario increased total daily mortalities by 0.11–0.27% from 2000s to 2050s [83]. For the same period Tagaris et al. [84] estimated that climate change under the SRES A1B scenario increased combined PM_2.5_ (long-term exposure) and O_3_ (short-term exposure) related annual U.S premature mortalities by 4,300 deaths. Examining climate policies that reduced the global mean temperature change from 6 °C to ~1.5 °C produced ~50,000 avoided premature mortalities for the USA [45]. Over Europe, Orru et al. [85], estimated annual premature mortalities due to short-term exposure to O_3_ to increase over most of Europe but decrease over the northernmost Nordic and Baltic countries, with the largest change being a 34% increase over Belgium under the SRES A2 climate scenario in the 2050s, due to regional reductions in cloud cover and soil moisture. In a sensitivity study over the UK, Heal et al. [86] found that a 5 °C increase of year-round temperatures increased the total UK health burden due to short-term exposure to O_3_ by an additional 500 premature deaths (4%), assuming no change in population and no threshold for O_3_ effects.

Most recently, examining the climate change impacts only from the RCP 8.5 scenario from an ensemble of nine chemistry-climate models participating in the Atmospheric Chemistry and Climate Model Intercomparison Project (ACCMIP), Silva et al. [87] estimated 43,600 (95% CI: -195,000 to 237,000) global respiratory-related deaths due to long-term ozone exposure, and 215,000 (95% CI: -76,100 to 595,000) global deaths in 2100 relative to 2000 due to long-term PM_2.5_ exposure. For Europe, 2,890 (95% CI: -4680 to 15,800) ozone respiratory-related deaths and 9850 (95% CI: 4550 to 16,100) PM_2.5_ deaths due to climate change alone were estimated in 2100 compared to 2000.

## Future climate and emission scenarios combined- air quality and health impacts

The attribution of future climate change on air quality can be difficult for several reasons: results are strongly influenced by the driving global climate model, simulations need to be of sufficient duration to separate climate change from climate variability, and the impacts of climate change on air pollution are likely to depend on the magnitudes of anthropogenic emission change [29, 82]. The majority of recent studies that consider both air-quality impacts from climate change as well as consequent health effects under the RCP scenarios typically consider combined emission and climate change, which are reviewed below. For the RCP scenarios, changes in global-mean temperature associated with changes in greenhouse gas emissions have been given in the previous section. All RCP scenarios assume strong abatement measures: NO_x_ emissions are reduced by ~50% in 2100 (from ~80 Tg NO yr.^−1^ to 30–50 Tg NO yr.^−1^) compared to 2000 levels and black carbon (BC) emission also reduce by a similar percentage (see Fig. 1; [29]). These measures generally result in large decreases in pollutant precursor species globally [29]. However, the CH_4_ abundance more than doubles in 2100 compared to 2005 for the RCP 8.5 scenario whilst RCP 2.6 predicts a ~25% reduction (Fig. 1; [29]).

From the ACCMIP models, Young et al. [11] suggest annual-mean O_3_ increases of >10 ppbv over Europe (see their Fig. 9) under RCP 8.5 emissions and climate in 2100 compared to 2000. Over Europe, Langner et al. [80] studied air quality in 2100 under the SRES A1B climate scenario together with O_3_ precursor emission changes from the RCP 4.5 (4.5 W m^−2^ net radiative forcing at 2100) scenario. They found that in southern Europe projected emissions reductions more than offset the climate penalty or the increase in April–September daily max O_3_. In northern Europe, both emission reductions and climate change decreased O_3_. Coleman et al. [88] reported similar findings for the RCP 6.0 scenario with the most significant changes after 2050 due to the pattern of changing emissions. However, by 2100, changes in meteorology were found to be important over the North Atlantic region.

**Fig. 3:**
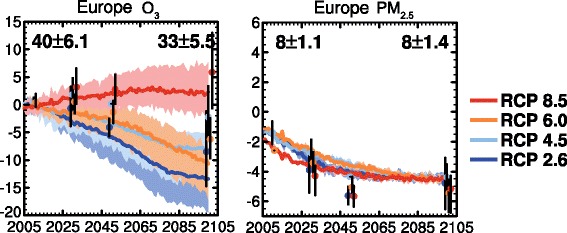
Adaptation (with permission) of Figures 11.23a and 11.23b from chapter 11, IPCC WG1 Fifth Assessment Report (Kirtman et al. [74]), showing European panels only. Projected changes in annual-mean (left) O_3_ (ppbv) and (right) PM_2.5_ (μg m^−3^) from 2000 to 2100 following the RCP scenarios (8.5 red, 6.0 orange, light blue 4.5, 2.6 dark blue) averaged over Europe (land). Coloured lines show the average and shading denotes the full range of 4 chemistry-climate models and coloured dots and vertical bars represent the average and full range of ~15 ACCMIP models for decadal time slices centred on 2010, 2030, 2050 and 2100. The average value and model standard deviation for the reference period is shown in the top of each panel for CMIP5 models (left) and ACCMIP models (right). For further details, see Kirtman et al. [74]

The combined effects of emission and climate changes under the RCP scenarios averaged over Europe based on IPCC AR5 [74] are shown in Fig. 3. By 2100, European multi-model annual-mean surface O_3_ changes between −15 to +2 ppbv relative to 2000 and PM_2.5_ changes between −4 to −6 μg m^−3^. The increase in O_3_ under RCP 8.5 reflects primarily the increase in CH_4_ emissions [29, 89], whilst the decreases in the other three scenarios reflect the role of emission reductions of other O_3_ precursors. PM changes depend on oxidant levels but generally follow SO_2_ emissions and primary organic carbon emissions reductions [29, 74]. All RCP scenarios suggest similar decreases in European-average PM_2.5_. Overall, the emissions changes strongly drive changes in O_3_ and PM_2.5_ in terms of annual-mean metrics. These are either augmented or reduced by the multiple effects of climate on atmospheric composition. However, as discussed above, for other metrics relating to peak exposure levels, the effects of changes in climate may be relatively more important.

Collette et al. [82] examined the effects of the RCP 2.6 and RCP 8.5 climate scenarios alongside compatible (but not RCP) emission scenarios on air quality in 2050 for both O_3_ and PM_2.5_. For both pollutants, they showed that the main factor driving future air quality projections for Europe was precursor emission changes, rather than climate change. A clear O_3_ climate penalty was found across most of Europe. The resulting change in population-weighted SOMO35 O_3_ health metric (defined as the annual sum of daily maximum 8 h running mean O_3_ over 35 ppbv) varied between +7% to −80% depending on the emission scenario whilst exposure-weighted PM_2.5_ was reduced by 62–78%. However, the authors point out the sensitivity to the emission scenarios used and to precipitation projections. Kim et al. [90] further note the potential of climate-related increases in natural NO_x_ emissions from lightning and soils to offset anthropogenic NO_x_ emission changes. Over the UK, greater O_3_ penalties from emissions changes compared to temperature change were also reported [86].

Most recently, Silva et al. [91] quantified premature morality related to O_3_ and PM_2.5_ long-term exposure, using ensemble-mean results from 14 ACCMIP models for the four RCPs for combined emissions and climate scenarios, additionally considering future population projections. Global excess O_3_-related (316,000 deaths/year in 2100) were found under the RCP8.5 but avoided O_3_-related respiratory mortalities (−718,000 to −1.02 million deaths/year) resulted from the other three RCP scenarios respectively, in line with their respective changes in O_3_ concentrations as described above. For RCP8.5, climate change was found to contribute 14% of the overall increase in global ozone mortality estimated in 2100 relative to 2000 [87]. Equivalent values for Europe were +2,390 (RCP 8.5) to −24,900 to −44,600 (other three RCP scenarios) annual avoided deaths in 2100. However, when the change in population between 2000 and 2100 was also considered, the global mortality burden of O_3_ increased from 382,000 deaths/yr. in 2000 to 1.1–2.4 million deaths/yr. in 2100 depending on RCP scenario [91]. Reduced global PM_2.5_ concentrations in all RCP scenarios led to global avoided premature mortalities ranging between −2.4 to −1.3 million deaths/yr. (for Europe: −103,000 to - 112,000 avoided premature mortalities in 2100) [91]. Under RCP 8.5, climate change countered the decrease in global PM_2.5_-related mortality by 16% in 2100 relative to 2000 [87]. Considering future PM_2.5_ concentrations and population change, the global mortality burden of PM_2.5_ decreased from 1.7 million deaths/yr. in 2000 to 0.95–1.55 million deaths/yr. in 2100 depending on RCP scenario [91]. Differences in simulated pollutant concentrations were highlighted as the major source of overall mortality uncertainty [87, 91].

## Key exposure-related determinants on future air quality health burdens

The extent of adverse health impacts from exposure to surface O_3_ depends markedly on whether a threshold concentration of O_3_ below which no impact is assumed. A recent review by COMEAP [3] suggest no evidence of a threshold or a counter-factual concentration other than zero, yet the WHO [7] does recommend the use of a threshold of 70 μg m^−3^ due to greater availability of data in the warm season when lower values than this threshold are less prevalent, outside of highly polluted regions.

There are also uncertainties in the appropriate magnitude of risk coefficient to use, and the part of the year for which O_3_ exposure is relevant for health impacts [7]. Uncertainties in risk estimates were suggested to have a similar or greater magnitude of influence as model uncertainty in simulated O_3_ concentrations for assessing the health effects of O_3_ and black carbon [92, 93].

Issues concerning potential modification of air pollution health effects by temperature remain pertinent for future climate-related assessments [23, 30, 94, 95]. In addition, daily baseline mortality and morbidity rates may not remain constant in the future. However, it is not possible to predict changes in risk coefficients or threshold effects due to adaptation to future pollutant levels or to future climate change [96]. Finally, the effects of higher air pollution levels and air pollution episodes will undoubtedly be modified by behaviour that affects exposure, such as time indoors and exercise that affects inhalation.

## Conclusions

There are numerous studies on future changes in air quality for surface O_3_ in particular, but very limited studies on the associated human health impacts. Globally, warming decreases background surface O_3_, but higher CH_4_ levels under the latest IPCC scenario RCP8.5 more than counteract this decrease, causing increased surface O_3_. Regionally, several studies suggest fairly robust climate change signals over Europe that lead to increases in summer-mean O_3_ in southern Europe and less change in northern Europe. The effects for higher concentration metrics such as daily maximum O_3_ are typically larger. For PM, studies related to climate change are still limited, and key uncertainties arise due to the differing effects of temperature on various PM components and uncertainties in future precipitation patterns. Further studies that examine the effects of climate change on PM would be beneficial.

There is consensus in the literature that, in the near term, air quality will be dominated by emission changes rather than changes in climate and/or long-range transport. However, for peak concentrations and longer future horizons the effect of the latter two processes may be much more relevant. The latest RCP scenarios show the strong influence of future CH_4_ levels on air quality. Controlling CH_4_ and possibly black carbon are viewed as win-win policies for mitigating air quality as well as climate change [97–99] and greenhouse gas policies have been related to human health benefits [100, 101].

Extreme air pollution episodes are associated with changing weather patterns, such as heat waves and stagnation episodes. A number of studies of the 2003 heatwave in Europe have identified various mechanisms associated with climate change that have a substantial effect on O_3_ and PM air quality. Air pollution episodes are more generally related to stagnation events which may in turn be related to larger-scale blocking and influenced by climate change. Studies that link changes in climate extremes to changes in air pollution characteristics (e.g., episode length and frequency, changes in high percentile values) are needed to quantify the effect of changes in climate extremes on air quality.

Key uncertainties in mechanistic understanding limit our confidence in future projections of air quality and air quality episodes. A major uncertainty for O_3_ and PM is the opposing influences of rising temperature and CO_2_ on natural isoprene emissions. The impact of climate change on transport pathways is also highly uncertain and model dependent. For example, projected changes in the frequency of regional air stagnation events remain difficult to assess.

Overall, the climate penalty effects on O_3_ and possibly PM indicates that stronger emission controls will be needed in the future to avoid higher health risks associated with climate change induced worsening of air quality in populated regions [50]. For O_3_-related health impacts, major uncertainties, besides model uncertainty in simulated O_3_ levels, are the impacts of long-term exposure, thresholds and effect modification by temperature. For PM-related health impacts, model-to-model variability in the response of PM_2.5_ concentrations to climate change seems likely to be the largest source of uncertainty. Spatial and temporal heterogeneity in risk estimates for O_3_ and PM are also an issue for modelling health impacts. Finally, there is uncertainty regarding future potential adaptation effects into the twenty-first century.

### Open peer review

Peer review reports for this article are available in Additional file 1.

## Additional file

Additional file 1: Open peer review. (PDF 161 kb)
