# Supplementary material for: Climate change impacts on human health over Europe through its effect on air quality
Source: Environ Health. 2017 Dec 5;16(Suppl 1):118. doi: 10.1186/s12940-017-0325-2 (PMC5773909; doi:10.1186/s12940-017-0325-2)
Supplement: Additional file 1: — Open peer review. (PDF 161 kb) [file 12940_2017_325_MOESM1_ESM.pdf]

Reviewer reports

Title: Climate change impacts on human health over Europe through its effect on air quality

Reviewer 1: Meridith Fry

This paper presents a review of the current literature on the impacts of climate change and future emissions on human health in Europe. The review is scientifically sound and based on numerous reputable sources. The authors provide an impartial, straightforward review of the evidence and also discuss the key uncertainties in current estimates. The paper appears appropriate for *Environmental Health*, as it examines how future climate and emissions changes may impact air quality and human health. Acceptance for publication after minor revisions is recommended.

Suggested revisions:

1) COMEAP should be defined as the Committee on the Medical Effects of Air Pollutants on pg. 2. The authors may also want to briefly describe why the evidence is not convincing COMEAP on pg. 2.
2) It would be helpful to list the main air pollutant precursors in Europe mentioned on pg. 3.
3) Figure 1 could be simplified or omitted. The caption does not seem to precisely describe the figure, and the figure may not be necessary. A bulleted list or outline of topics covered in the review would be clearer.
4) In Figure 2, the variable and impacts in each box could be designated more clearly. For example, each variable could be in bold, and a subheading, "Impacts:", could be used on the next line to separate the list of impacts from each variable.
5) In Figure 3, the caption should also include what the dotted bars represent.

Competing interests: I declare that I have no competing interests.

Reviewer 2: Darrell A. Winner

This review examines the current literature on the effects of future emissions and climate change on PM and O3 air quality and on the consequent health impacts, with a focus on Europe. I recommend publication, after revisions based on review.

Major comments:

1) The paper should be updated with several significant, recent publications in this field. Also, the references to North American studies seem uneven. This paper does cite some of the significant North American work, but excludes many more studies. Do the authors have selection criteria for the studies they include or exclude?

2) The abstract needs a major overhaul to better express the impact of climate change and to increase the detail and substance of statements in the abstract. The conclusion section is superior in language and approach compared to the abstract. I suggest using a similar approach in the abstract.

Specific comments on the Abstract

>"O3 averaged over Europe is expected to decrease under all scenarios due to higher water vapour concentrations, except under RCP8.5 when higher methane (CH4) emissions more than offset this decrease. In polluted areas with high levels of nitrogen oxides (NOx), elevated surface temperatures and humidities are likely to increase surface O3."

I am concerned that the lack of detail and order of these statements could be misleading. If the authors want to feature the decrease in background levels of ozone due to high water vapour concentrations, they need to appropriately discuss that this contrasts with the increases in the areas where most people live. I also disagree with construction of the first sentence here. Don't use "all scenarios" and then follow with the exception.

>"Future changes in PM concentrations due to climate change remain uncertain, due to the differing effects of temperature on various PM components and uncertainties in future rainfall projections."

This sentence is not an adequate summary of the good discussion later in the paper of future changes in PM concentrations. Please revise to better reflect the state of the (complex) science.

>"A number of studies have examined both future climate and emissions changes and there is some consensus that the impact of emission changes on air quality out to the 2050s will outweigh that due to climate change."

And also "When future emissions and climate change are considered together global O3 (except in RCP 8.5) and PM- related mortalities decrease."

I object to the contest framing here comparing changes in air quality due to climate change and changes in air quality due to future emission changes. The scientific question is how will air quality respond to a changing climate and to changing emissions. The result that (large) future emission reductions will lower air pollution does not provide insight into the magnitude of the climate penalty. This problem is exacerbated by the lack of details in the paper regarding the emission changes. For example, what are

some of the possible policies and regulations to achieve the emission levels of RCP4.5 vs. RCP8.5? Similarly, I do not see the value in the statement about decreasing mortalities under scenarios with both a changing climate and future (large) emission reductions. It would be more straightforward to report a quantitative expression of the climate penalty in both cases.

>"Several modelling studies suggest that elevated temperatures will also increase peak O3 and PM concentrations but there is large variability in the patterns and incidences of these events across the studies."

Can the authors make this statement more quantitative? How large are the ozone and PM increase? What does "large variability" mean?

>"There are very limited studies on health effects associated with climate change impacts on air quality,"

Again, can the authors provide more substance here? What do they mean by "very limited studies"?

Comments on other sections

Introduction:

>"There is now strengthened evidence for effects of long-term exposure to O3 on respiratory and cardiorespiratory mortality (WHO, 2013a), although taken as a whole this evidence not convincing COMEAP(2015)."

The end of this sentence is missing words. I assume the intent is to state that there is less evidence for O3 effects on mortality than PM effects on mortality. While this statement is certainly true, the scientific body of evidence for PM effect on mortality is robust and consistent across many studies. Is this the standard to judge the effects of other pollutants?

Review, current health burdens and risk estimates

>This section would be more readable if many of the risk values could be moved into a table. This could also enable more space to discuss the results, rather than using the text to report the results.

Future climate scenarios and air quality impacts:

>"There have been only a few studies to date examining the effect of climate change alone on human health burdens."

Judging from the following text, this sentence should be modified to include "human health burdens from air quality."

Competing interests: I declare that I have no competing interests.

Author response

We thank both reviewers for their endorsement of the appropriateness of our paper for publication in Environmental Health and for their constructive comments that have improved this manuscript. We provide our point by point responses and corresponding text changes/additions below. We hope that the revised manuscript can now be published in Environmental Health.

We have also provided updates in several places:
(i) We have updated the text in "current health burdens and risk estimates" with text from the later EEA 2016 and GBD 2015 reports/articles and appropriate references.
(ii) Updated references for articles in press.
(iii) In the section "Future climate and emissions scenarios combined" updated text describing results in Silva et al. (2016) to include values of premature mortalities for Europe.

Reviewer 1:

This paper presents a review of the current literature on the impacts of climate change and future emissions on human health in Europe. The review is scientifically sound and based on numerous reputable sources. The authors provide an impartial, straightforward review of the evidence and also discuss the key uncertainties in current estimates. The paper appears appropriate for Environmental Health, as it examines how future climate and emissions changes may impact air quality and human health. Acceptance for publication after minor revisions is recommended.

Suggested revisions:

6) *COMEAP should be defined as the Committee on the Medical Effects of Air Pollutants on pg. 2. The authors may also want to briefly describe why the evidence is not convincing COMEAP on pg. 2.*

This is a good point. The text on page 2 has been revised to: "There is now strengthened evidence for effects of long-term exposure to O_3 on respiratory and cardiorespiratory mortality in warm season months (WHO, 2013a), although taken as a whole this evidence is limited at present, as it is primarily derived from studies in North America (Committee on the Medical Effects of Air Pollutants: COMEAP; 2015)."

7) *It would be helpful to list the main air pollutant precursors in Europe mentioned on pg. 3.*

These have been added to page 3 with the appropriate reference; the text on page 3 now reads: "Emissions of the main air pollutant precursors: primary PM, black carbon (BC); nitrogen oxides (NO_x); sulphur dioxide (SO_2), carbon monoxide (CO), methane (CH_4) and non-methane volatile organic compounds (NMVOCs) in Europe have declined in the last decade or so, resulting in improvements in air quality across the region (EEA 2016)."

8) *Figure 1 could be simplified or omitted. The caption does not seem to precisely describe the figure, and the figure may not be necessary. A bulleted list or outline of topics covered in the review would be clearer.*

Figure 1 has been removed. The text in the following sentences describe the sections covered in the review. Text on page 3 revised to: “The topics covered in this review are as follows. First, current…”

9) *In Figure 2, the variable and impacts in each box could be designated more clearly. For example, each variable could be in bold, and a subheading, “Impacts:”, could be used on the next line to separate the list of impacts from each variable.*

This is an excellent idea- Figure 1 (previously Figure 2) has been revised as suggested.

10) *In Figure 3, the caption should also include what the dotted bars represent.*

Thank you for noting this omission. The Figure 2 (formerly Figure 3) caption has now been changed to the following: “Changes in surface O_3 (ppb) between year 2000 and 2030 driven by climate alone (CLIMATE; green) or emissions alone following CLE (black), MRF (gray), SRES (blue) and RCP (red) emission scenarios. Bars represent multi-model standard deviation with the exception of the green dotted line over Europe, which represents the range of climate-only changes in summer daily maximum O_3 from a single-model study (Forkel and Knoche, 2006). This is Figure 11.22 from Kirtman et al. (2013). For further details, see Kirtman et al. (2013).”

Reviewer 2

This review examines the current literature on the effects of future emissions and climate change on PM and O3 air quality and on the consequent health impacts, with a focus on Europe. I recommend publication, after revisions based on review.

Major comments:

3) *The paper should be updated with several significant, recent publications in this field. Also, the references to North American studies seem uneven. This paper does cite some of the significant North American work, but excludes many more studies. Do the authors have selection criteria for the studies they include or exclude?*

We have now included results from two recent studies on climate change effects on PM air pollution by Garcia-Menendez et al. (2015) and Allen et al. 2016 (see additional text below); the latter was published in 2016 after this paper was submitted. We have also included results from Garcia-Menendez et al. (2015) in relation to health impacts associated with changing climate policy.

In terms of providing results that links to climate change and health impacts, as we state in the abstract and introduction our focus is on Europe. This is because this is one of a series of papers on climate change impacts in the UK for this special issue. Since much of our material draws on global/regional model studies, Europe seemed the appropriate size of region on which to focus. For clarity, however, we have amended the title to: “Climate Change Impacts on Human Health over Europe through its effect on Air Quality”. We refer to North American studies to set the scene or describe a relevant climate-chemistry process or interaction where European studies do not yet exist or are limited. We have revised and added new text in the section “Future climate scenarios and air quality impacts as follows to include results from these two papers:

“Most recently, several studies have suggested a PM climate penalty in the future (Garcia-Menendez et al. 2015; Allen et al. 2016). A PM climate penalty simulated in 2050 and 2100 in the eastern USA was attributed to enhanced sulphate concentrations with higher temperature and humidity (Garcia-Menendez et al. 2015). A recent multi-model study suggested that climate change, simulated under the RCP 8.5 climate scenario, increases the aerosol burden and surface PM concentrations, through a reduction in large-scale precipitation over northern mid-latitude land regions (Allen et al. 2016). Over Europe, one regional modelling study reported the geographical patterns of the impact of climate on surface summer PM levels to be less robust than for O_3 (Collette et al. 2013). “

“Examining climate policies that reduced the global mean temperature change from 6° C to ~1.5°C produced ~50,000 avoided premature mortalities for the USA (Garcia-Menendez et al. 2015).”

4) *The abstract needs a major overhaul to better express the impact of climate change and to increase the detail and substance of statements in the abstract. The conclusion section is superior in language and approach compared to the abstract. I suggest using a similar approach in the abstract.*

We have revised the abstract extensively as well as all relevant sections of the main text in accordance with the specific points raised below, referring to our conclusions for guidance in revising the abstract as suggested.

Specific comments on the Abstract

>“O3 averaged over Europe is expected to decrease under all scenarios due to higher water vapour concentrations, except under RCP8.5 when higher methane (CH4) emissions more than offset this decrease. In polluted areas with high levels of nitrogen oxides (NOx), elevated surface temperatures and humidities are likely to increase surface O3.”

I am concerned that the lack of detail and order of these statements could be misleading. If the authors want to feature the decrease in background levels of ozone due to high water vapour concentrations, they need to appropriately discuss that this contrasts with the increases in the areas where most people live. I also disagree with construction of the first sentence here. Don’t use “all scenarios” and then follow with the exception.

We agree that the text should be clearer. The abstract text has been revised to separate out the climate change impact on background versus regional ozone. The text now reads:

”Under the latest Intergovernmental Panel on Climate Change (IPCC) 5th assessment report (AR5) Representative Concentration Pathways (RCPs), background O_3 entering Europe is expected to decrease under most scenarios due to higher water vapour concentrations in a warmer climate. However, under the extreme RCP8.5 pathway higher methane (CH4) emissions leads to increases in background O_3 that offset the O_3 decrease due to climate change. Regionally, in polluted areas with high levels of nitrogen oxides (NOx), elevated surface temperatures and humidities yield increases in surface O_3 especially in southern Europe. The O3 response is larger for metrics that represent the higher end of the O3 distribution, such as daily maximum O3.”

>"Future changes in PM concentrations due to climate change remain uncertain, due to the differing effects of temperature on various PM components and uncertainties in future rainfall projections."

This sentence is not an adequate summary of the good discussion later in the paper of future changes in PM concentrations. Please revise to better reflect the state of the (complex) science.

We agree. This sentence now reads: "Future changes in PM concentrations due to climate change are much less certain, although several recent studies also suggest a PM climate penalty due to high temperatures and humidity and reduced precipitation in northern mid-latitude land regions."

We have also included the recent literature by Garcia-Menendez et al. 2015 and Allen et al. 2016 as outlined in our response to the major comments above.

>"A number of studies have examined both future climate and emissions changes and there is some consensus that the impact of emission changes on air quality out to the 2050s will outweigh that due to climate change."

And also "When future emissions and climate change are considered together global O3 (except in RCP 8.5) and PM- related mortalities decrease."

I object to the contest framing here comparing changes in air quality due to climate change and changes in air quality due to future emission changes. The scientific question is how will air quality respond to a changing climate and to changing emissions. The result that (large) future emission reductions will lower air pollution does not provide insight into the magnitude of the climate penalty. This problem is exacerbated by the lack of details in the paper regarding the emission changes. For example, what are some of the possible policies and regulations to achieve the emission levels of RCP4.5 vs. RCP8.5? Similarly, I do not see the value in the statement about decreasing mortalities under scenarios with both a changing climate and future (large) emission reductions. It would be more straightforward to report a quantitative expression of the climate penalty in both cases.

It is not possible to report a climate penalty effect in the combined climate and emission change studies we refer to above. However, we agree that our text is imprecise, hence we have revised text in the abstract and added new text to the relevant sections of the body of the text. The studies to which we refer in these sentences have considered the combined effect of greenhouse gas emissions influencing climate and pollutant-precursor emissions changes and generally do not separate out the climate penalty alone but report the overall impact as given above. Hence, a quantitative expression for the climate penalty is not possible. In addition, we have reported quantitative changes in the text but not in the abstract as these values vary greatly depending on the model used, the time period and the pollutant metric used- an important detail which it is not possible to include within the confines of an abstract. We have revised these two sentences above in the abstract to read more clearly and precisely:

"A larger number of studies have examined both future climate and emissions changes under the RCP scenarios. Under these pathways the impact of emission changes on air quality out to the 2050s will be larger than that due to climate change, because of large reductions in emissions of O_3 and PM pollutant precursor emissions and the more limited climate change response itself."

"Studies that examine the combined impacts of climate change and anthropogenic emissions change under the RCP scenarios report reductions in global and European premature O_3-respiratory related and

PM mortalities arising from the large decreases in precursor emissions. Under RCP 8.5 the large increase in CH_4 leads to global and European excess O_3-respiratory related mortalities in 2100."

In addition, we have added to the main text details regarding the levels of climate change in terms of global mean temperature change and of pollutant precursor emission changes (as requested above). These have been added to the start of the relevant sections of the text on "future climate scenarios and air quality impacts" and "future climate and emissions scenarios and impacts." We are unable to address specific policies that could lead to the different RCP scenarios as they were designed top- down to achieve a radiative forcing without a specific policy in mind. The additions are as follows:

"The global–mean temperature change projected with Global Climate Models (GCMs) driven by the SRES climate scenarios is between 1.4-6.3°C in the 2090s. GCM projections of global average warming using the RCP climate scenarios is between 0.3-1.7˚C for RCP2.6 and 2.6-4.8˚C for RCP8.5 in 2100 compared to 1986-2005 (IPCC, 2013)."

"The majority of recent studies that consider both air-quality impacts for climate change as well as consequent health effects under the RCP scenarios typically consider combined emission and climate change, which are reviewed below. For the RCP scenarios changes in global-mean temperature associated with change in greenhouse gas emissions have been given in the previous section. All RCP scenarios assume strong abatement measures: NOx emissions are reduced by ~50% in 2100 (from ~80 Tg NO yr-1 to 30-50 Tg NO yr-1) compared to 2000 levels and Black Carbon (BC) emission also reduce by a similar percentage (see figure 1; Fiore et al. 2012). These measures generally result in large decreases in pollutant precursor species globally (Fiore et al. 2012). However, the methane (CH_4) abundance more than doubles in 2100 compared to 2005 for the RCP 8.5 scenario whilst RCP 2.6 predicts a ~25% reduction (figure 1; Fiore et al. 2012)."

>"Several modelling studies suggest that elevated temperatures will also increase peak O3 and PM concentrations but there is large variability in the patterns and incidences of these events across the studies."

Can the authors make this statement more quantitative? How large are the ozone and PM increase? What does "large variability" mean?

This text mainly comes from Kirtman et al. (2013) but we have not discussed this in more detail in the main body of the text hence we have removed this sentence. However we have added the following sentence to the conclusions where extremes and episodes are mentioned:

"Studies that link changes in climate extremes to changes in air pollution characteristics (e.g., episode length and frequency, changes in high percentile values) are needed to quantify the effect of changes in climate extremes on air quality."

>"There are very limited studies on health effects associated with climate change impacts on air quality,"

Again, can the authors provide more substance here? What do they mean by "very limited studies"?

We have revised the abstract text to be in line with the main body text as much as possible:

“There are few studies on health effects associated with climate change impacts alone on air quality, but these report higher O_3-related health burdens in polluted populated regions and greater $PM_{2.5}$ health burdens in these emission regions.”

Comments on other sections

Introduction:

>“There is now strengthened evidence for effects of long-term exposure to O3 on respiratory and cardiorespiratory mortality (WHO, 2013a), although taken as a whole this evidence not convincing COMEAP(2015).”

The end of this sentence is missing words. I assume the intent is to state that there is less evidence for O3 effects on mortality than PM effects on mortality. While this statement is certainly true, the scientific body of evidence for PM effect on mortality is robust and consistent across many studies. Is this the standard to judge the effects of other pollutants?

This text on page 3 has been revised as follows (as revised in response also to a comment by Reviewer 1):

“There is now strengthened evidence for effects of long-term exposure to O_3 on respiratory and cardiorespiratory mortality in warm season months (WHO, 2013a), although taken as a whole this evidence is limited at present, as it is primarily derived from studies in North America (Committee on the Medical Effects of Air Pollutants: COMEAP; 2015).”

There are several other studies in other regions that have found non-significant relationships between long-term ozone exposure and premature mortality: (Atkinson et al. 2016; Bentayeb et al. 2015; Carey et al. 2013). However for space reasons we have not added these to the text as we were already at the word limit requested.

Atkinson RW, Butland BK, Dimitroulopoulou C, Heal MR, Stedman JR, Carslaw N, et al. 2016. Long-term exposure to ambient ozone and mortality: a quantitative systematic review and meta-analysis of evidence from cohort studies. BMJ Open 6:e009493–e009493; doi:10.1136/bmjopen-2015-009493.

Bentayeb M, Wagner V, Stempfelet M, Zins M, Goldberg M, Pascal M, et al. 2015. Association between long-term exposure to air pollution and mortality in France: A 25-year follow-up study. Environ. Int. 85:5–14; doi:10.1016/j.envint.2015.08.006.

Carey IM, Atkinson RW, Kent AJ, van Staa T, Cook DG, Anderson HR. 2013. Mortality Associations with Long-Term Exposure to Outdoor Air Pollution in a National English Cohort. Am. J. Respir. Crit. Care Med. 187:1226–1233; doi:10.1164/rccm.201210-1758OC.

Review, current health burdens and risk estimates

>This section would be more readable if many of the risk values could be moved into a table. This could also enable more space to discuss the results, rather than using the text to report the results.

This is an excellent point. Table 1 now contains risk estimates as requested and the text has more discussion. The text in this section now reads:

“Dose-response coefficients used to quantify the risk of mortality related to short and long-term exposure to $PM_{2.5}$ and O_3 are given in Table 1. These coefficients are based on the Health Risks of Air Pollution In Europe (HRAPIE) project (WHO 2013b) and results from long-term American Cancer Society (ACS) cohort studies (Krewski et al. 2009; Jerrett et al. 2009). For long-term exposure to $PM_{2.5}$, WHO (2013b) suggest an increased premature mortality risk of 6.2% per 10 µg m^{-3} exposure measured using annual-mean $PM_{2.5}$ concentrations (Table 1). For short-term exposure to O_3, an increased premature mortality risk of 0.29% per 10 µg m^{-3} exposure measured using daily maximum 8-hour running mean O_3 is suggested by WHO (2013b) (Table 1); they also recommend a threshold of 70 µg m^{-3} due to greater data availability for the warm season. “

We have also updated text in the conclusions on threshold recommendations based on this revised text.

Future climate scenarios and air quality impacts:

>“There have been only a few studies to date examining the effect of climate change alone on human health burdens.”

Judging from the following text, this sentence should be modified to include “human health burdens from air quality.”

The text now reads: “There have been only a few studies to date examining the effect of climate change alone on human health burdens from air quality.”

Reviewer reports – 2nd round

Reviewer 1: Meridith Fry

The authors' responses have addressed my comments and suggestions. I recommend the revised manuscript for publication.

Reviewer 2: Darrell A. Winner

Yes, the authors addressed my comments satisfactorily. I recommend publication of the revised manuscript.
